# Infrared skin temperature measurements for monitoring health in pigs: a review

**DOI:** 10.1186/s13028-015-0094-2

**Published:** 2015-02-03

**Authors:** Dennis Dam Soerensen, Lene Juul Pedersen

**Affiliations:** Department of Animal Science, Aarhus University, Blichers Allé 20, PO Box 50 , DK-8830 Tjele, Denmark

**Keywords:** Infrared thermography, Temperature, Fever, Thermoregulation, Pig

## Abstract

Infrared temperature measurement equipment (IRTME) is gaining popularity as a diagnostic tool for evaluating human and animal health. It has the prospect of reducing subject stress and disease spread by being implemented as an automatic surveillance system and by a quick assessment of skin temperatures without need for restraint or contact. This review evaluates studies and applications where IRTME has been used on pigs. These include investigations of relationships between skin, ambient and body temperatures and applications for detecting fever, inflammation, lesions, ovulation, and stress as well as for meat quality assessment. The best skin locations for high correlation between skin temperature and rectal temperature are most likely thermal windows such as ear base, eye region and udder. However, this may change with age, stressors, and biological state changes, for example, farrowing. The studies performed on pigs using IRTME have presented somewhat discrepant results, which could be caused by inadequate equipment, varying knowledge about reliable equipment operation, and site-specific factors not included in the assessment. Future focus areas in the field of IRTME are suggested for further development of new application areas and increased diagnostic value in the porcine and animal setting in general.

## Introduction

Infrared thermography (IRT) and infrared thermometry (IRTM) are non-contact temperature measurement methods, which offer several advantages over other temperature measurement methods used in veterinary medicine. Infrared (IR) temperature measurements considerably reduce the risk of spreading infection, since touching the subject is unnecessary. In animals, this is advantageous since handling and restraint increases stress, causing an effect on core and surface temperatures [1-7]. Due to the non-obtrusive nature of IRTME, it is an insignificant stressor, theoretically making it well-suited for assessment of animal stress and welfare. The potential of IRTME is reflected in the various applications for which it has been used. IRT was used successfully for detecting meat defects [8-12] and has been attempted for predicting estrus from vulva surface temperature [13-15]. Others have used IRTM [16] and IRT [17,18] in feed and digestion studies to evaluate its suitability for, aiding or replacing calorimetry. IRTME does not affect the temperature of the object surface by conduction or convection, which is the case with regular temperature sensors. IRT acquires thermal images, enabling measurement of surface temperature distributions, suited for detecting localized anomalies. It is possible to select regions of interest (ROIs) in the thermal images, thereby only selecting specific areas of interest for subsequent data analyses. These ROIs may be analyzed by spots (e.g. single pixel), lines, or areas of various shapes. While IRT produces thermal images with many pixels, IRTM utilizes only one pixel, which measures the average temperature of the area it covers.

IRT is fitting for measuring skin temperature in pigs, since pelage of pigs is different than many other mammals, which have more hair coverage. Even though pig hair is much coarser than human hair, hair density is comparable to that of humans [19], offering several bare skinned surface areas.

Reviews covering the use of IRTME on animals [20-24] have had very little focus on pigs. The aim of this review is to assemble studies investigating use of IRTME on pigs with focus on the temperature relationships between environment, skin, and body as well as detection of fever and other health issues.

### Surface temperature in relation to ambient temperature

Thermoregulatory ability in pigs increases with age. As opposed to a piglet having less than 2% body fat during the first two weeks of life [25], an adult pig has a thicker fat layer, a denser hair coat and a smaller surface to volume ratio, enabling them to limit the heat flow from the body core to the surroundings. From here on, body core temperature is referred to as body temperature. Piglets maintain their body temperature by reducing the exposure to the colder environment, for example, by huddling and by increasing their heat production by shivering [26-28]. Due to their lack of insulation, newborn piglets may be looked upon as one large thermal window, where the skin temperature is almost uniform throughout the body surface. A thermal window is a skin area, which is always well perfused by blood and as such is a “window” to the body temperature [29]. This in opposition to a non-thermal window, such as the ear flap or shoulder, where blood perfusion to the outer skin surface is controlled by thermoregulation. From here on, when referring to measured skin temperatures, they have been measured by IRT or IRTM, if not mentioned otherwise.

IRTME measures the surface temperature of pigs. The surface temperature of the skin (hairy or bare) is influenced by environmental ambient factors and the thermoregulatory response by the body that maintains a stable body temperature. Therefore, if an elevated skin temperature due to fever is to be detected correctly, it is necessary to quantify the effect of ambient temperature on skin temperature for non-febrile pigs. Ultimately, this should be included in a correction formula, which could be used for fever determination purposes. The few studies that have investigated this relationship are summarized in Table 1. These studies vary in their approach regarding the area of skin, age, breed, experimental setting and use of different IRTME and methodology. This makes it impossible to combine the findings into one correction formula. However, studies have shown that skin areas with an increased fat layer (non-thermal window), which may be breed dependent, have a lower regression constant and higher coefficient [30]. This means that skin temperature is lower at low ambient temperatures due to the fat insulation, while increasing ambient temperature rapidly increases the skin temperature, due to the vasocontrolled thermoregulation, supplying more blood to the skin tissue. As expected, of the studies included in Table 1, the thermal windows exhibit higher regression constants and lower regression coefficients than the non-thermal windows, as they are less affected by the ambient room temperature.

**Table 1:** **Studies showing relationship between surface (measured by infrared devices) and ambient temperature in healthy pigs**

**Source**	**N**	**Breed**	**Approx. weight (kg)**	**Surface area(s)**	**Thermal Window (X: yes)**	**T**_**ambient**_ **[°C]**	**Regression Constant, T**_**0**_ **[°C] (** ^**A**^ **)**	**Regression coefficient, b (** ^**A**^ **)**	**Correlation (r) and goodness of fit (R** ^**2**^ **) (** ^**A**^ **)**	**Remarks**
Henken *et al*. [30]	16	Norwegian, Finnish and Dutch Landrace and Great Yorkshire	26	Lumbal area		11-26	22.5–25.9	0.37–0.47	r = 0.83–0.91 (**)	(^B^)
Wendt *et al*. [34]	89		7–60	Ear base	X	12–30	28.5	0.263	r = −0.68 (***)	(^C^)
	147		160–222				27.6			
Loughmiller *et al*. [2]	4	Crossbred	30	Loin		10–32	24.8	0.40	R^2^ = 0.97 (***)	
Collin *et al*. [35]	8	[Large White × Landrace] × Piétrain	15–35	Interscapula region		23–33	29.3	0.29	(**)	(^B^)
Savary *et al*. [36]	9		50–70	Ear base, abdomen (caudal) and upper medial side of legs	X	12–18	30.6	0.36	(***)	
				Carpus, tarsus, shoulder, knee and elbow			25.2–27.1	0.48		
Costa *et al*. [37]	12		8	Part of body with the highest temperature	X	22–36	30.8	0.20	R^2^ = 0.44 (***)	
			11		X	16–28	33.7	0.20	R^2^ = 0.57 (***)	
Malmkvist *et al*. [31]	39	Danish Landrace × Yorkshire	>200	Eye	X	15–25	32	0.2	(***)	(^B^)
				Snout			28.7	0.3		
				Udder (caudal)	X		32	0.2		

^A^T_skin_ = T_0_ + b*T_ambient_ , where T_0_ = regression constant [°C], b = regression coefficient ( = T_skin_ increase per 1°C increase in T_ambient_). **:*P* < 0.01 and ***:*P* < 0.001.

^B^Estimations performed by author of this article.

^C^Based on thermocouple temperature measurements, not IR.

### Surface temperature in relation to core body temperature

Knowing the skin temperature for non-febrile pigs under different ambient temperatures may not be enough for measuring elevated body temperature, which is the most important indicator of fever. A prerequisite for detecting elevated body temperature by measurement of skin temperature is a sufficient correlation between the two. Table 2 summarizes the studies that have investigated the correlation between skin surface temperatures measured using IRTME and body temperature. Usually, the body temperature remains relatively constant due to thermoregulation, but in certain situations, body temperature is regulated or altered: fever, hypothermia, digestion, parturition, and diurnal rhythm. Body temperature is usually determined by inserting a lubricated thermometer into the rectum, with the probe touching the mucus lining. Penetration depth depends on size of the pig, but in an adult sow, the probe should be inserted approx. 10 cm into the rectum. See Table 3 for normal rectal temperatures in resting pigs of different age at ambient temperatures in the thermoneutral zone.

**Table 2:** **Studies showing relationship between surface (measured by infrared devices) and body temperature in pigs**

**Source**	**N**	**Breed**	**Size/Age**	**Surface area(s)**	**Thermal window (X: yes)**	**T**_**ambient**_ **[°C]**	**Correlation (r) (** ^**A**^ **)**	**Remarks**
Zinn *et al*. [33]	15	York × Landrace (LR) × Duroc	225 kg	Eyeball	X	N/A	0.14 → 0.51	Before → after parturition. Hairy sites were trimmed.
				Ear (outer side, 5 cm from base)	X		0.12 → 0.39	
				Tail head			0.23 → 0.29	
				Shoulder			0.18 → 0.47 (*)	
				Flank			0.28 → 0.42	
				Perineum	X		0.21 → 0.30	
				Vulva	X		0.16 → 0.23	
				Udder (cranial)	X		0.06 → 0.27	
				Udder (middle)	X		0.12 → 0.44	
				Udder (caudal)	X		0.13 → 0.39	
Loughmiller *et al*. [2]	24	Crossbred	30 kg	Loin		21 ± 2	0.52 (***)	
Warriss *et al*. [7]	384		~91 kg	Inner side of ear pinna	X^B^	21	0.71 (***)	(^C^)
Traulsen *et al*. [32]	42	Crossbred	1st–6th parity	Eyes	X	20.5–24.3	0.33 (*)	
				Udder	X		0.41 (*)	
				Inner ear	X		0.31 (*)	
				Ear base	X		0.32 (*)	
				Vulva region (lying)	X		0.49 (*)	
				Vulva region (standing)	X		0.32 (*)	
Magnani *et al*. [4]	108		10–17 days	Eye	X	22–30	0.02	
				Side, back and womb	X^B^		0.02–0.33	
				Axilla	X		0.33	
Chung *et al*. [38]	10	Yorkshire × LR (Gnotobiotic)	Piglets	Central abdomen	X	21 ± 1	0.58 (***)	
				Central dorsum			0.30 (***)	
				Perianal region	X		0.43 (***)	
Malmkvist *et al*. [31]	39	Danish LR × Yorkshire	1st–3rd parity	Snout		15–25	0.21 (**)	(^D^)
				Eye	X		0.49 (***)	(^D^)
				Udder (caudal third)	X		0.60 (***)	(^D^)
Sykes *et al*. [14]	32	Yorkshire × LR	~85 kg	Vulva (estrus)	X	1.5–25.8	−0.099 to 0.011	
				Vulva (diestrus)	X		−0.115 to −0.068	
Tabuaciri *et al*. [39]	485	Large White (LW) × LR, LW × Duroc	<2 kg	Ear base	X	N/A	0.85	
				Ear tip			0.27	
				Back (crown to rump)			0.75	

^A^If only the coefficient of determination (R^2^) was provided in the article, the magnitude of the correlation (r) between body and IR measured skin temperature was calculated by taking the square root of R^2^. *:*P* < 0.05, **:*P* < 0.01, ***:*P* < 0.001.

^B^Indicates measurement sites that are both thermal windows and not thermal windows.

^C^Temperature was the mean of the 3 spots of the ear (base, middle, and tip). Body temperature was measured by aiming IR spotmeter on blood on concrete floor, lost from sticking neck vessels.

^D^Numbers provided from personal communication.

**Table 3:** **Normal rectal temperatures in resting pigs in the thermoneutral zone. Modified from** [40]

**Age**	**Normal rectal temperature,°C (variation ± 0.3°C)**
Piglets	39.5
Slaughter pigs	39.3
Young sows, gilts	38.8
Multiparous sows	38.3

### Correlation between skin and body temperature

The limited amount of studies found in the literature that investigated the correlation between skin and body temperature include a range of ages from newborn piglets to old sows. Furthermore, the biological status of the investigated pigs was different, for example, with sows being investigated around the time of farrowing [31-33], slaughter pigs just killed with carbondioxide and exsanguinated in a stressful abattoir [7], and growing pigs being inoculated with *Actinobacillus pleuropneumoniae* [2]. This makes it difficult to compare the correlation between skin and body temperature even between the same skin areas. However, there are indications that the skin measurement sites for highest correlation to body temperature are the ears, eyes and udder. One study [32] found that the skin areas with the highest repeatability were the eyes and ears, even though the vulva skin area (when lying down) exhibited the highest correlation (r = 0.49) to body temperature. The standing position reduced the correlation substantially (r = 0.32), which is difficult to explain physiologically and may emanate from differences in methodology. In contrast, another study [14] did not find any correlation between vulva surface temperatures and body temperature, but a significant correlation between the vulva surface temperature and ambient temperatures was observed. This may be explained by the limited acclimatization period (30 min.) and moisture in the vulva orifice, causing cooling by evaporation, since within the ROI, the minimum temperature was 20.6°C and the maximum was 35.6°C.

The correlation between the ear skin and body temperature seems highly dependent on the exact location of which the skin temperature was measured. The ear flap is an important thermoregulatory area, while other areas such as the ear base and ear canal area may be considered thermal windows. This may explain the discrepant results reported [4,7,32,33,39] and is nicely highlighted in the study by Tabuaciri *et al*. [39], who found a much higher correlation between the body and skin temperature of the ear base (r = 0.85) compared to the ear tip (r = 0.27). The study by Warriss *et al*. [7] differed from the other studies by the method used for measuring body temperature (see Table 2). Their reported high correlation (r = 0.71) could be due to the stunning damage to the pigs’ brains, altering their vasocontrolled thermoregulation and the stressful situation in which the measurements took place (a commercial abattoir). Stress is known to have a significant effect on the surface temperatures of the ear and eyes [4] and the loin [2] in pigs. The initial vaso-constrictive response decrease the skin temperature, while after some time, this may subside, followed by an increase in skin temperature, releasing heat built up in the core from the initial vasoconstriction [2]. Similar results were found in rabbits [3] and cattle [41-43]. A low correlation coefficient (r = 0.02) between the eye and body temperature was observed in response to a stressor (a backtest) in pigs [4]. Other studies investigating the correlation between eye and body temperature reported higher correlation coefficients, which has also been reported in humans [44-46] and ponies [47]. Perhaps a pig study focusing specifically on the inner canthus, with adequate equipment (high spatial resolution and accuracy) and using the maximum pixel temperature would reveal higher correlation, as seen in humans [44-46]. The inner canthus of the eye was chosen as the best site for fever screening in humans [48,49] due to the relatively easy access and its high correlation to body temperature. However, the high correlations reported were often based on tympanic temperature as the reference for body temperature and one study questions the suitability of the inner canthus to estimate body temperature [50], while another suggests the ear canal as a potentially better site [51]. Regardless, if the maximum temperature is to be detected in such relatively small areas or orifices as the inner canthus and the ear canal opening, the demand for a high spatial resolution is essential. Use of maximum or near maximum pixel temperatures for body temperature estimation in pigs is further supported by Traulsen *et al*. [32], who found that the maximum, average of the top-10% or top-25% IRT pixel temperatures in sows yielded higher body temperature correlation than average or minimum pixel temperatures for all the investigated surface areas (see Table 2).

A few studies performed a regression analysis of the skin - body temperature relationship in pigs (summarized in Table 4). Even at similar ambient temperatures, the studies reveal notably different regression constants and coefficients. This may be due to the limited variation in body temperature, even when observed animals were febrile. Furthermore, accuracy of the rectal thermometer measurements may be influenced by the experience of the operator and this may account for the variability between measurements. The only study that did not use rectal measurements for measuring the body temperature was also one of the studies with the highest regression coefficient [7]. They measured body temperature from blood lost from a sticking wound. However, with the large range of the measured body (blood) temperatures (35.6–42.6°C), the results are questionable. Another explanation for the discrepant results in Table 4 (and Table 2) may be due to the skin - body temperature relationship not being linear, at least for some skin areas. Malmkvist *et al*. [31] found an increased correlation coefficient (r = 0.32) for the snout if using a quadratic relationship vs. a linear (r = 0.21) and Traulsen *et al*. [32] found a significant quadratic relationship between the skin and body temperatures in all investigated skin areas other than the vulva and eyes (see Table 2).

**Table 4:** **Studies that investigated the linear regression between surface (measured by infrared devices) and body temperature**

**Source**	**N**	**Breed**	**Size/Age**	**Surface area(s)**	**Thermal window (X: yes)**	**T**_**ambient**_ **[°C]**	**Regression (** ^**A**^ **)**		**Remarks**
							**Constant, T**_**0**_ **[°C]**	**Coefficient, b**	
Dewulf *et al*. [54]	12	Belgian LR	12–15 kg	Ear		~20	38.80	0.015	
				Flank			37.82	0.044 (**)	
				Legs (carpus and tarsus)			38.24	0.032 (**)	
				Anus	X		38.02	0.038 (**)	
Warriss *et al*. [7]	384		~91 kg	Inner side of ear pinna	X^B^	21	4.93	1.12 (***)	r = 0.71 , (^C^)
Chung *et al*. [38]	10	Yorkshire × LR (Gnoto-biotic)	Piglets	Eyelid, ear, parietal and axilla	X^B^	21 ± 1			No significant relationship
				Central abdomen	X		28.07	0.304 (***)	Adj. R^2^ = 0.34
				Central dorsum			34.03	0.152 (***)	Adj. R^2^ = 0.09
				Perianal region	X		33.88	0.157 (***)	Adj. R^2^ = 0.19
Kammersgaard *et al*. [55]	91	(LR × Yorkshire) × Duroc	1–48 hours	Entire piglet from dorsal view and sagittal view	X^B^	15, 20, and 25	14.72	0.63 (***)	Avg. of max. IR pixel temp. from dorsal view and sagittal view

^A^Regression formula: T_body_ = T_0_ + b*T_skin_. Body temperature measurement site was the rectum if not otherwise mentioned. **:*P* < 0.01 and ***:*P* < 0.001.

^B^Indicates measurement sites that are both thermal windows and not thermal windows.

^C^Temperature was the mean of the 3 spots of the ear (base, middle, and tip). Body temperature was measured by aiming IR spotmeter on blood on concrete floor, lost from sticking neck vessels.

### Detection of fever in pigs

Fever, as determined by rectal temperature, has been shown to elevate the skin temperature significantly in pigs, although with an expected time delay [2]. However, the skin temperature does not increase equally over the entire pig surface. Traulsen *et al*. [32] observed average skin temperature increases in sows, ranging from 0.20°C for the vulva to 0.56°C for the inner ear and up to 0.99°C for the udder.

Johnson and Dunbar [52] reported a skin temperature increase 6 days post inoculation (classical swine fever) of 5.8, 12 and 10°C for the edge of ear, foot and entire body surface, respectively. They also observed the maximum temperature of the entire body surface was found inside the hind legs and on the dorsum of the pig. Similar results have been reported in pigs infected with the *A. epizooticae* virus (foot and mouth disease) with maximum foot skin temperature elevating by more than 10°C three days after infection [53].

Dewulf *et al*. [54] also investigated pigs with classical swine fever and deemed skin temperature measured using IRTM as insufficient for fever detection, since no reliable rectal temperature could be predicted, even though a significant correlation was observed (see Table 4). Wendt *et al*. [34] also investigated the diagnostic power by measuring ear base skin temperature, obtained by using regular thermocouple sensor put against skin. The sensitivity and specificity for sows were 79.0%, 69.3%, respectively, with positive (healthy) and negative (febrile) predictive values of 78.5% and 70.0%, respectively. Similarly for young pigs the numbers were 55.8%, 93.6%, 84.0% and 77.7%, respectively. Schmidt *et al*. [56] were able to detect 6 and 7 febrile sows out of 10, from eye and ear base temperature, respectively, where fever threshold values were based on 90% quantiles. Most of the diagnostic performance numbers presented in the mentioned studies are on the low side and may not be satisfactory in most settings. This may pertain to the delay between increase of skin and body temperature from febrile onset as observed in cattle [57].

One investigation used IRT for pen-level fever detection in pigs [58]. Instead of investigating the individual pigs for fever, the maximum temperature measured in pens with approx. 20 pigs was used for detection of pens with *Actinobacillus pleuropneumoniae* infection. Pens with mortality had significantly higher maximum pen temperature compared to three control pens (with no mortality) on the day that the mortality was observed, and the two days before. With each increase by 1°C in maximum pen temperature, the odds of a pen having mortality increased 3.6 times. The concept of using the group of pigs as its own control by comparing to *a priori* temperature measurements, was investigated further in another study [59], which found thermal response to a vaccination using a surveillance system.

### Detection of inflammation and lesions

Sabec and Lazar [60] showed that boars with palpable symptoms of osteoarthrosis tarsi deformans had significant increased average skin temperature (36.27°C) of the surrounding skin surface when compared to boars without palpable symptoms (35.64°C). Savary *et al*. [36] showed that IRT enabled detection in 40% of leg joints with proven acute inflammation, but only 9.5% of leg joints with chronic inflammation. With their diagnosis criteria, IRT indicated approx. 10% of leg joints in healthy pigs to be inflamed. They observed large temperature differences (>1°C) between symmetrical regions in more than one fourth of the leg joint measurements in healthy pigs, questioning reliability of this method for detecting inflammation based on asymmetry alone. The measured asymmetries in healthy pigs of >1°C are high compared to the max of 0.5°C found in healthy humans [61,62]. This discrepancy could be explained by the housing facilities in which pigs rest. One could speculate that hard floors would cause elevated temperatures on the surface areas bearing the weight of the pig, causing major changes in the subdermal tissue, as is seen in sows with decubital pressure ulcers [63].

Active dynamic thermography (ADT) is a method by which IRT recordings following an external heat or cold exposure may reveal differences in the underlying tissue of the surface in question due to different recooling or reheating patterns. Thermal properties of pig skin have been shown to change when burned [64,65]. In these studies, pig skin was used for modeling human skin and they showed that ADT performed just as well as the histopathological method (reference), in evaluating skin burn depth, whereas the clinical method (using traditional observational criteria) and static IRT performed worse. The ADT and hisopathological methods performed equally, exhibiting 100% accuracy, sensitivity, and specificity. For the clinical method, these 3 numbers were 60.9%, 50%, and 100%, respectively, while for the static IRT method, the numbers were 91.3%, 94.4%, and 80.0%, respectively. ADT has also been found to be superior compared with static IRT when characterizing wound types and assessing pressure ulcers [66].

### Improving infrared measurements and their applications

#### Fever diagnosis

Obviously, we cannot claim that IRT should be the new gold standard for detecting fever in pigs from the diagnostic test results reported in the literature.

There is a great demand for a comprehensive study using calibrated, accurate IRTME investigating pigs from different age groups (with known fat layer), at different skin areas, and at a varying range of ambient temperatures. Findings from this could result in correction formulae (linear or quadratic), which could be used in a number of different settings, where variation in ambient temperature, age, and skin location could be accounted for. Ultimately, this would then be used as a rough baseline value; from which discrepancies could be attributed, e.g. fever. Furthermore, including ADT in future diagnostic tests may reveal that this method is not only suited for detecting localized skin damage or inflammation [64,65], but also for elevated systemic temperatures.

Adopting techniques as proposed by Ng and Kee [67] could further improve diagnosis. Ng and Kee [67] used a combination of parabolic regression, learning algorithms and receiver operating characteristics (ROC) analysis instead of the conventional method consisting of linear regression and ROC analysis. In doing so, they increased the accuracy of diagnoses in fever screenings of humans. Accuracy rate for the same measurements was increased from 93% to 96%, while sensitivity and specificity changed from 85.4% and 95% to 95% and 85.6%, respectively.

#### Standardization protocols

The diagnostic toolbox additions as mentioned above may be insufficient for improving fever detection from IRT measurements. If the skin measurements themselves are inaccurate, fever diagnosis will inevitably be inaccurate. However, todays advanced IR cameras provide fairly accurate temperature measurements with good spatial and thermal resolution. Furthermore, diagnoses based on skin measurements using IRT could be improved considerably by creation of standardization protocols as have been made for fever screening in humans [48,49,68]. Operators of IR cameras should be trained properly, knowing the limitations of the equipment [69]. This could reduce the variability in temperature measurements due to operator errors, e.g. wrong choice of skin emissivity or incorrect measurement of reflection temperature [70]. In addition to this, introducing the Digital Imaging and Communication in Medicine (DICOM) standard in the field of IRT in general as suggested by some scientists [71,72] will be a valuable addition. If used correctly, this will enable researchers and scientists to compare IRT measurements performed with different devices by different operators knowing that factors like distance, reflections, angles, emissivity, poorly calibrated devices, etc. have been taken into consideration for best possible measurement results. Obviously, thermal properties between animal species and breeds are different due to several factors, such as body temperature set points, thermoneutral zone differences, thermal windows, and hair density. These factors should be considered when comparing any temperature measurements.

## Conclusions

Further research on use of IRT for pig health screening is still needed before it can be used as a reliable diagnostic tool. Studies performed to date are inconclusive due to much variance in results. Most of which are likely to arise from inaccurate measurements and methodology differences. IRT shows great potential in some health applications where it may become superior to other methods. However, ground research incorporating metrological approaches for accurate measurements is necessary to make IRT a reliable standardized method for detection of changes in body surface temperatures due to fever, inflammation, stress, pain or other conditions.

Thorough investigations of surface temperature distributions of healthy pigs of different age groups, breeds and in different environments is the first step towards improving detection of unhealthy pigs. Furthermore, skin temperature progression at different stages in the course of fever for various diseases should be made. This could enable detection of sick animals prior to evidence of clinical symptoms.
